# Effect of food simulating liquids on the flexural strength of a methacrylate and silorane-based composite

**DOI:** 10.1371/journal.pone.0188829

**Published:** 2017-12-12

**Authors:** Ehsan Mohammadi, Leila Pishevar, Parvin Mirzakouchaki Boroujeni

**Affiliations:** Department of Operative Dentistry, Faculty of Dentistry, Isfahan (Khorasgan) Branch, Islamic Azad University, Isfahan, Iran; Ondokuz Mayis Universitesi Dis Hekimligi Fakultesi, TURKEY

## Abstract

**Objective:**

The purpose of this study was to assess the effect of food-simulating liquids on the flexural strength of a methacrylate-based and a silorane-based resin composite.

**Materials and methods:**

In this in vitro study, sixty specimens of Filtek P90 and Filtek Z350 composite were prepared in a customized mold (2 × 2 × 25 mm). The specimens of each composite were divided into five subgroups as follows: one as a control group and the other four groups included distilled water, heptane, 2% citric acid, and 50% aqueous ethanol. The specimens were stored in the solutions for one week at 37°C, and the control group was stored at room temperature for the same period of time. Then, flexural strength values were measured. The statistical analysis was performed by One-Way ANOVA, Paired T test and post hoc LSD at a significance level of 0.05.

**Results:**

In the control group, the mean flexural strength of Filtek P90 and Filtek Z350 were 155.1 MPa and 147.3 MPa, respectively, and there was no significant difference (P-value>0.05). The mean flexural strength of Filtek P90 and Filtek Z350 significantly decreased in ethanol (P-value <0.05). Immersion in 0.02 N citric acid and heptane had no significant effect on the flexural strength of Filtek P90 and Filtek Z350. The maximum flexural strength of Filtek P90 was in the heptane group (192.6 MPa) and minimum flexural strength was in ethanol group (92.7 MPa) the maximum flexural strength of Filtek Z350 was in the heptane group (163.2 MPa) and minimum flexural strength was in the ethanol group (104.7 MPa).

**Conclusion:**

The flexural strength of tested resin composites significantly affected by ethanol solution. The flexural strength of resin composites was not affected by other food simulating liquids.

## Introduction

Resin composites are widely used in modern dentistry today. A wide range of composite materials including conventional composites, such as hybrid, nanofilled, silorane, Ormocer, and Compomer, are available for direct restorations. The differences between these materials regarding the type of monomer, filler composition, and the chemical structure of binding agent of the filler matrix (silane) have led to differences in mechanical characteristics of these materials resulting in differences in the resistance of the composites against mechanical forces and chemical damage [1].

There are different types of damages that occur to resin composites. It could be a result of matrix and filler destruction, because of mechanical forces and debonding, tiny cracks, or breakage of particles, which leads to the reduced durability of composite resin restorations in clinical conditions [2]. In an oral environment, the resin composites are intermittently or continuously exposed to chemical compositions of saliva, food, and drinks. Organic acids and their many derivatives and food-like fluids can cause softening of the resin matrix of the composite resin restorations. This degradation occurs because of two reasons: hydrolytic breakdown of the bond between the silane and the filler–resin matrix that would eventually lead to the debonding and softening of resin through water absorption [3]. The effect of organic acids and food simulating liquids on some surface characteristics of composite resins with a methacrylate resin base such as wear, hardness, and surface roughness have been investigated in several studies [4–7]. However composite resin based on silorane was not recently launched in the market. Silorane-based composites contract less during their polymerization process, which occurs through the opening of monomeric oxirane rings. It is also claimed that these composites are insoluble and stable in biological fluids [8]. Thus, the aim of this study was to investigate the effect of food simulating liquids on the flexural strength of a methacrylate and a silorane-based composite. The null hypothesis of the study is that the food simulating liquids affect the flexural strength of methacrylate and silorane-based composite similarly.

## Materials and methods

The details of the materials utilized are given in Table 1. In this in vitro study, 60 specimens of Filtek Z350 and Filtek P90 (3M ESPE, Seefeld, Germany) were prepared using a split stainless steel mold (2 × 2 × 25 mm) according to ISO 4049: 2000 specification [9]. Initially, the inside surface of the mold was slightly lubricated with Vaseline, and then, the resin composite was placed inside the mold in single increment. Then, a glass slide was placed on it and pressed to remove the excess material. A light meter was used to standardize the output of the light curing device (LITEX 695 C, Dent America, USA), and the samples were light-cured in two stages, each time for 40 seconds with 1000 mw/cm^2^ intensity (in the first stage, the two light cure units were placed together, and in the second phase, the remaining parts of the samples were cured with one light curing device). Then, the mold was opened and the specimen was removed. The top surfaces of the specimens were marked to distinguish it from bottom surfaces. Thereafter specimens were measured with a digital caliper. The specimens were randomly divided into four test groups and one control group (n = 6). The randomization procedure was carried out by using sequentially numbered opaque sealed envelope prepared with unrestricted (simple) randomization. Each treatment agent was written and sealed in envelopes before beginning the study. The Control specimens were stored for a week at room temperature in a light proof box, and the other groups (distilled water, heptane, 0.02 N citric acid and 50% aqueous ethanol) were incubated for one week at a temperature of 37°C. After one week, the samples were washed with water and air-dried. For determination of the flexural strength a three-point bending test was conducted according to ISO 4049. After mounting in the Instron universal device (K-21046, Walter-bai, Switzerland), the specimens were loaded to failure in such a way that the force would be applied to the center of composite beams using a crosshead speed of 1 mm/minute. The distance between the supports was 20 mm. The maximum force applied until the breakage of the specimens was recorded and the flexural strength (S) was calculated in MPa according to the formula:

$$3FL/2BH^{2}$$

Where F is the maximum force applied in Newton, L is the distance between the supports, B is the width of the samples and H is the height of the samples. The values were measured.All the calculations were carried out using SPSS 18.0 for Windows (SPSS Corporation, Chicago, IL, USA). Mean and standard deviations were calculated. Normal distribution of data was verified. The data obtained were subjected T Paired test, one-way ANOVA and post hoc LSD.

**Table 1:** Composition of the materials tested.

Material	Composition
Filtek P903M ESPE, Seefeld- Germany	Bis- 3,4 Epoxycyclohexylethyl-Phenyl-Methylsilane 3,4Epoxycyclopolymethyisiloxane Silanized, Quartz, Ytterium fluoride (0.1–2 μm, 55 vol %)
Filtek Z350 3M ESPE, Seefeld-Germany	Bis- GMA, UDMA, TEGDMA, PEGDMA, Bis-EMA, Siloxan, Sillica(20nm), Zirconia(4-11nm), Silica/Zirconia Cluster Filler (63.3 vol%)

## Results

**Fig 1:**
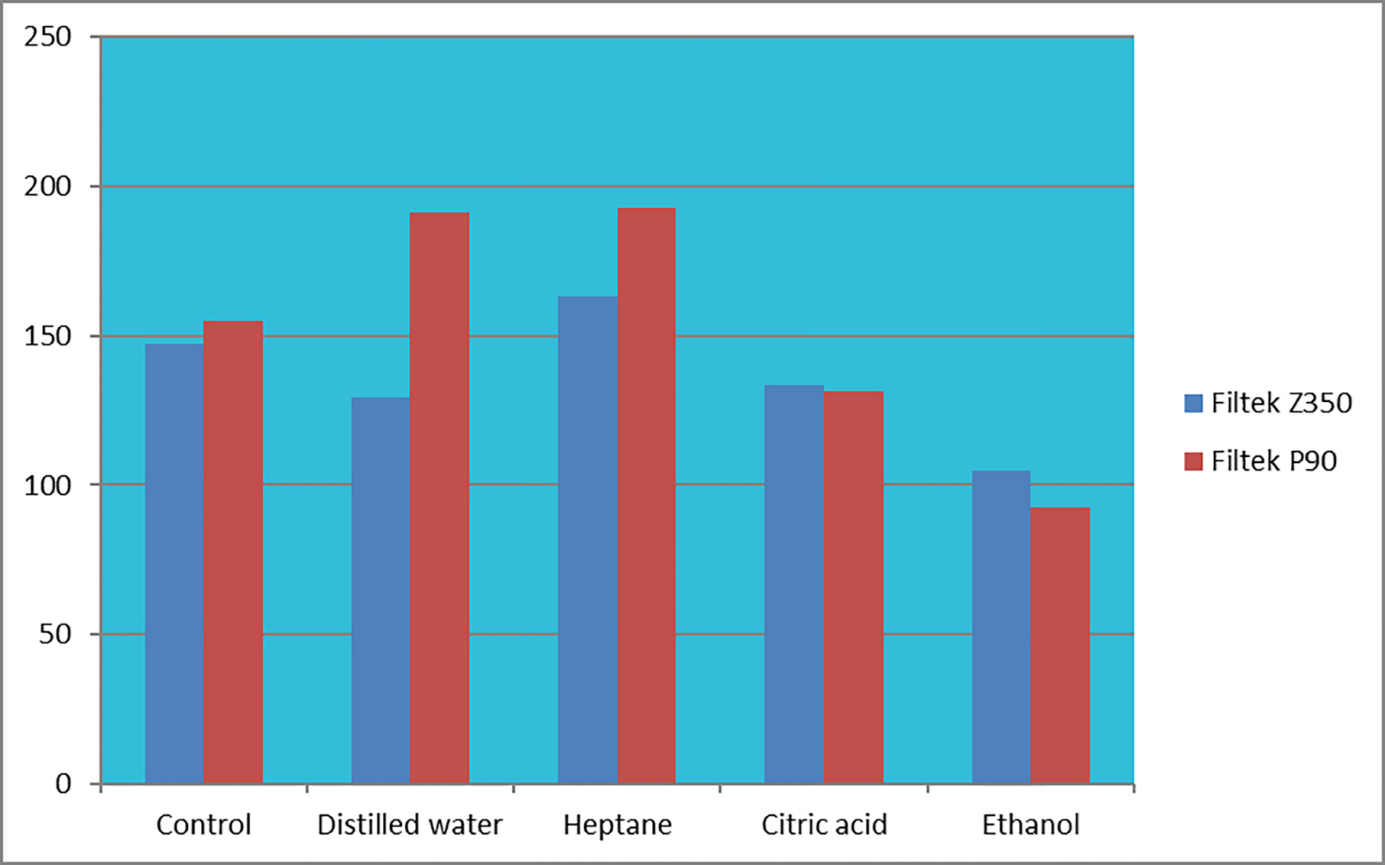
Mean flexural strength value for the tested materials.

The averages of the flexural strength of 10 experimental groups are shown in Table 2 and Fig 1. No significant differences between the Filtek P90 and Filtek Z350 was in the control group for the average flexural strength (p-value >0.05). The statistical test also showed that in distilled water, the average flexural strength of Filtek P90 was significantly higher than the average flexural strength of Filtek Z350 (p-value<0.05), but the averages of flexural strength of the Filtek P90 and Filtek Z350 in other solutions were not significantly different. The average flexural strength of both Filtek P90 and Filtek Z350 significantly reduced in the 50% aqueous ethanol solution (p-value<0.05). The one-way ANOVA test revealed that the average flexural strength of Filtek P90 was not the same in different groups (p-value <0.05). As can be seen in Table 2, the maximum and minimum flexural strength were related to the heptane group and ethanol group, respectively. The test also showed that the mean flexural strength of the Filtek Z350 was not identical in different groups (p-value< 0.05). The complete results are given in Table 2.

**Table 2:** Means (standard deviations) of flexural strength value [Mpa] for the materials tested.

**Medium**	Filtek Z350	Filtek P90
**Control**	147.3 (15.09)	155.1 (47.7)
**Distilled water**	129.6 (37.8)	191.1 (66.9)
**Heptane**	163.2 (27.4)	192.6 (45)
**Citric Acid**	133.2 (13.2)	131.4 (52.5)
**Ethanol**	104.7* (16.5)	92.7* (19.8)

* Statistically Significant difference (P < 0.05)

## Discussion

In previous years, improved mechanical properties of resin-based material associated with increased demand in esthetic components have led to the widespread use of resin composites in anterior and posterior restorations [4]. Although many physical and mechanical properties of composites have been improved over the time, polymerization shrinkage is still known as one of the weaknesses of the composites. Silorane has been introduced as an alternative for methacrylate in the composite matrix because of its low polymerization shrinkage and its hydrophobic property. Despite considerable progress in composition and properties, the composites must still withstand adverse conditions in the oral environment, which challenge their strength and integrity [10]. Some chemicals in foods and beverages can cause damage to the composite, which leads to damage to the appearance, increased surface roughness, and rapid wear of material [10, 11]. The matrix of resin-based composites is susceptible to softening by various organic acids, foods, and drinks, whereas it is claimed that the silorane-based composites are stable and insoluble in biological fluids [2]. Composite restorations are exposed continuously and intermittently to various chemical attacks in the oral environment. Intermittent exposure to chemical agents occurs during eating or drinking and continues until the mouth is cleaned of these substances. On the other hand, continuous exposure may occur when the chemical agents are absorbed through debris at the margins of restoration or produced by the bacterial decomposition of debris [12].

Chemical compounds are trapped around the margins of inadequately finished restorations providing conditions for the long contact of composite restorations with chemicals. Various chemicals exposed continuously or intermittently to the composites will affect their surface characteristics as well as physical-mechanical properties [13]. One of these mechanical properties is flexural strength. Flexural strength, also called as transverse strength, is the strength of a rod placed on two supports while subjected to static force [14]. This feature plays an important role in servicing and clinical survival of the restorations, which has a particular importance for the long-term performance of the restoration. Therefore, we sought to investigate the effect of food simulating liquids on this feature. Previous investigations reported that the maximum effect of these materials on the surface properties of the composites occurs during the first 7 days of immersion [15]. For this reason, a 7 days storage period designed for the present study. Samples of two types of silorane-based and methacrylate-based were placed in the solutions of distilled water, heptane, 50% aqueous ethanol, and 0.02 N citric acid for a week. The null hypothesis of the present study was accepted. The results revealed that the flexural strength of composites in control groups had no significant differences, a finding consistent with other studies conducted on the flexural strength of these two groups of composites [2, 16, 17]. However, some other studies have reported that the flexural strength of silorane-based composites is higher than the methacrylate-based composites [18]. One reason for the difference in the flexural strength between the composites could be the difficulty in preparation of a suitable and ideal specimen from the composites to perform flexural strength testing so that it is influenced by the shape, size, and maintenance conditions of the specimen [19]. The effect of each of the food-like solution on the flexural strength is examined separately.Flexural strength after immersion in distilled water showed no significant changes for both Filtek P90 and Filtek Z350. Yesilyurt et al. also showed that distilled water had no significant effect on the flexural strength of silorane-based and methacrylate-based composites [2]. In this study, the distilled water only led to a reduction in the flexural strength of the methacrylate-based composite. The methacrylate-based composites typically consist of a resin matrix, glass or ceramic filler, and a bonding agent of filler and matrix [20]. According to reports, increased TEGDMA in the resin matrix would lead to increased water uptake, since this monomer has more hydrophilic properties compared to Bis-GMA and UDMA [21]. It seems that the hydrophilic properties of TEGDMA monomer, which leads to greater absorption of water in this monomer, will influence the mechanical properties of methacrylate-based composites while having no effect on their flexural strength. In the present study, a 7-day immersion of specimens in 50% aqueous ethanol solution significantly reduced the flexural strength of both Filtek P90 and Filtek Z350 composites. This finding is consistent with the results of a study by Yesilyurt [2]. The studies conducted on the effect of oral fluids on the stability of the composites are designed to investigate the aging process by exposing the composites to water, artificial saliva, and solutions of ethanol/water at different compositions including 75% and 50% [22]. The effects of these chemical compounds are different, but they typically include washing out of the non-reacted monomeric components and a destructive effect on the polymeric network [23, 24]. The organic solvents absorbed by the polymeric network of the methacrylate-based composites are a few percent of its overall weight. This network does not dissolve but begins to swell when it is placed in a suitable solvent as long as the forces of attraction between the polymer chains are higher than the attraction forces between the solvent molecules and the chain components. Thus, the variation resulting from the interaction of secondary bonds will lead to an increased volume of the network, and softening will occur because of reduced interaction between the chains and enhanced plasticity. Softening is the separation of polymer chains by a molecule that does not form a primary chemical bond with the chain, but acts as a space-occupying agent. Thus, the main effect of the solvent is to reduce the interaction between the chains. The softening rate is consistent with the rate of solvent absorption, which starts immediately and reaches its maximum rate within one or two months as the network is fully saturated by the solvent. In a study by Vouvoudi, 75% aqueous ethanol solution had a greater effect on the mechanical properties of the composites compared to water and artificial saliva, which may be because of the organophilic nature of ethanol. Ethanol causes softening and degradation of the polymer matrix and eliminates the bonding between the filler and the silane [22].

According to Mckinney investigations the resin matrix of the composites could be potentially damaged by organic solvents, including heptane [25]. Additionally, the destruction of inorganic filler particles contributes to reducing the mechanical properties [26]. In the present study, the 7-day storage of Filtek P90 and Filtek Z350 in heptane solution increased their flexural strength; but this was not a significant increase. The studies have shown different results regarding the effect of heptane on the surface and mechanical characteristics of the composites. In the study by Yap et al., maintaining the methacrylate-based composite of Silux in heptane led to significant increase in stiffness of the composite [12]. In the study by Yesilyurt et al, heptane increased the hardness of methacrylate-based composites of Filtek P60, Filtek Z250, and Filtek Supreme, although the increase was not statistically significant [2]. A similar relationship has been reported by other studies [27, 28]. However, in the study by Yap and Low, a significant increase was observed in the hardness of a methacrylate-based composite of Silux [4]. There are two possibilities that could explain this. Firstly, reduced formation of the inhibiting oxygen layer by heptane and inhibiting the release of silica filler, and secondly it could be due to metals combined with filler particles in organic solvents such as heptane [29].

Examining the effect of immersion in citric acid, it was shown that the flexural strength of both silorane-based (Filtek P90) and methacrylate-based (Filtek Z350) composites reduced after 7 days of immersion in comparison to the control group, although the reduction was not statistically significant. In general, the possible harmful effects of intraoral weak acids (citric acid and lactic acid) may be observed through their effects on inorganic fillers [13]. In a study by Yesilyurt, the hardness of the silorane-based and methacrylate-based composites also showed no significant changes after immersion in citric acid for a week [2]. However, in the study by Akova, citric acid significantly reduced the flexural strength of some of the methacrylate-based material [13]. In addition, lactic acid, a weak organic acid similar to citric acid, led to decreased hardness of composites in a study by Yap et al [5]. Weak organic acids such as citric acid and lactic acid have a damaging effect on inorganic fillers that could possibly influence the reduction in flexural strength of the composite [25]. It was shown in the present study that citric acid led to decreased flexural strength in both Filtek P90 and Filtek Z350 composites, although the decrease was not significant. If the composites were kept in citric acid for longer, the difference may become statistically significant. Besides, the adverse effect of acids depended on pH and citric acid is a weak organic acid with a low acidic concentration.

## Conclusions

Based on the results mentioned above, the flexural strength of siloran and methacrylate based composites are simillarly affected by exposure to food substances. Ethanol significantly decreased the flexural strength of both tested composites. Significant change was not found following heptane and citric acid immersion.

## Supporting information

S1 File Statistical analysis as output.pdf file. (PDF)

S2 File Statistical analysis as output.spv file. (RAR)
