# Supplementary material for: Effect of food simulating liquids on the flexural strength of a methacrylate and silorane-based composite
Source: PLoS One. 2017 Dec 12;12(12):e0188829. doi: 10.1371/journal.pone.0188829 (PMC5726734; doi:10.1371/journal.pone.0188829)
Supplement: S1 File — (PDF) [file pone.0188829.s001.pdf]

Descriptives

		N	Mean	Std. Deviation
p90	control	6	155.1122	47.71201
	water	6	191.1431	66.92310
	heptan	6	192.6460	45.00123
	sitric acid	6	131.4045	52.51235
	ethanol	6	92.7354	19.83424
	Total	30	152.8474	71.23030
z350	control	6	147.3012	45.27131
	water	6	129.6235	113.40373
	heptan	6	163.2001	82.26404
	sitric acid	6	133.2433	39.66402
	ethanol	6	104.7460	49.53869
	Total	30	131.6521	133.61319

ANOVA

		Sum of Squares	df	Mean Square	F	Sig.
p90	Between Groups	8460.036	4	2115.009	4.557	.024
	Within Groups	11603.085	25	464.123		
	Total	20063.121	29			
z350	Between Groups	2904.697	4	726.174	3.540	.042
	Within Groups	5128.344	25	205.134		
	Total	8033.041	29			

Post Hoc Tests

Multiple Comparisons

LSD

Dependent Variable	(I) group	(J) group	Mean Difference (I-J)	Std. Error	Sig.
p90	control	water	-36.07500	37.31448	.173
		heptan	-37.47000	37.31448	.163
		sitric acid	23.62500	37.31448	.265
		ethanol	62.36499	37.31448	.048
	water	control	36.07500	37.31448	.173
		heptan	-1.39500	37.31448	.970
		sitric acid	59.70000	37.31448	.049
		ethanol	98.43999*	37.31448	.014
	heptan	control	37.47000	37.31448	.163
		water	1.39500	37.31448	.970
		sitric acid	61.09500	37.31448	.045
		ethanol	99.83500*	37.31448	.013
	sitric acid	control	-23.62500	37.31448	.265
		water	-59.70000	37.31448	.049
		heptan	-61.09500	37.31448	.045
		ethanol	38.73999	37.31448	.153
	ethanol	control	-62.36499	37.31448	.048
		water	-98.43999*	37.31448	.014
		heptan	-99.83500*	37.31448	.013
		sitric acid	-38.73999	37.31448	.153
z350	control	water	17.51500	24.80727	.245
		heptan	-15.97000	24.80727	.265
		sitric acid	14.08000	24.80727	.292
		ethanol	42.57000	24.80727	.042
	water	control	-17.51500	24.80727	.245
		heptan	-33.48500	24.80727	.082
		sitric acid	-3.43500	24.80727	.891
		ethanol	25.05500	24.80727	.163
	heptan	control	15.97000	24.80727	.265
		water	33.48500	24.80727	.082
		sitric acid	30.05000	24.80727	.112
		ethanol	58.53999*	24.80727	.013
	sitric acid	control	-14.08000	24.80727	.292
		water	3.43500	24.80727	.891
		heptan	-30.05000	24.80727	.112
		ethanol	28.49000	24.80727	.133

Multiple Comparisons

LSD

Dependent Variable	(I) group	(J) group	Mean Difference (I-J)	Std. Error	Sig.
	ethanol	control	-42.57000	24.80727	.042
		water	-25.05500	24.80727	.163
		heptan	-58.53999*	24.80727	.013
		sitric acid	-28.49000	24.80727	.133

*. The mean difference is significant at the 0.05 level.

T-Test

Paired Samples Statistics

group			Mean	N	Std. Deviation	Std. Error Mean
control	Pair 1	p90	155.1122	6	47.71201	19.49256
		z350	147.3012	6	45.27131	6.15873
water	Pair 1	p90	191.1431	6	66.92310	27.27314
		z350	129.6235	6	113.40373	15.38507
heptan	Pair 1	p90	192.6460	6	45.00123	42.80983
		z350	163.2001	6	82.26404	34.48883
sitric acid	Pair 1	p90	131.4045	6	52.51235	21.43298
		z350	133.2433	6	39.66402	5.34120
ethanol	Pair 1	p90	92.7354	6	19.83424	8.06770
		z350	104.7460	6	49.53869	6.77239

Paired Samples Test

group			Paired Differences			t	Sig. (2-tailed)
			Mean	Std. Deviation	Std. Error Mean		
control	Pair 1	p90 - z350	7.77500	56.01722	22.86894	.340	.748
water	Pair 1	p90 - z350	61.36500	69.33281	28.30500	2.168	.042
heptan	Pair 1	p90 - z350	29.27500	172.68547	70.49855	.415	.352
sitric acid	Pair 1	p90 - z350	-1.77000	41.43098	16.91413	-.105	.921
ethanol	Pair 1	p90 - z350	-12.02000	25.98054	10.60651	-1.133	.308
